# Giant Pericardial Lipoma Inducing Cardiac Tamponade and New Onset Atrial Flutter

**DOI:** 10.1155/2020/6937126

**Published:** 2020-03-02

**Authors:** Connor Charles Kerndt, Alexander Michael Balinski, Hayk Vahe Papukhyan

**Affiliations:** ^1^Michigan State University College of Osteopathic Medicine, East Lansing, MI 48824, USA; ^2^Oakland University William Beaumont School of Medicine, Rochester, MI 48309, USA; ^3^Department of Internal Medicine, Henry Ford Macomb Hospital, Clinton Township, MI 48038, USA

## Abstract

Although pericardial lipomas are both rare and benign, rapid or excessive growth can induce potentially fatal conditions such as pericarditis, arrhythmia, and cardiac tamponade. This case illustrates an example where a 65-year-old with atypical chest tightness unveiled a 10 × 15 cm anterior pericardial mass with circumferential effusion and progressive deterioration to cardiac tamponade. Initial transthoracic echocardiogram imaging was technically difficult in this patient due to habitus and body mass, which failed to illustrate underlying effusion. Recurrent bouts of refractory supraventricular tachycardia prompted further investigation of this patient's presentation with transesophageal echocardiogram, which showed evidence of an echogenic mass with cardiac tamponade. An urgent pericardial window and pericardial lipectomy immediately relieved this hemodynamically compromising condition. Subsequent atrial flutter resulted with the removal of the anterior fat pad during surgery, complicating recovery.

## 1. Introduction

Pericardial tumors are exceptionally rare, composing only 6.7-12.8% of all primary cardiac neoplasms [1, 2]. Cardiac tumors can present asymptomatically with incidental findings on imaging or arise with a myriad of symptoms dependent on location, size, and subtype. Pericardial lipomas are benign pericardial neoplasms with a predominance of adipose cells which generally grow insidiously but can expand to compress and irritate adjacent structures via mass effect [3]. Rarely, they can induce fluid infiltration into the pericardial sac due to inflammatory mechanisms, resulting in pericarditis [4].

## 2. Case Presentation

A 65-year-old female presented to the emergency department with pleuritic chest pain, tachycardia, and eructation. The chest pain was characterized as a “band-like” chest tightness that radiated to the left axilla accompanied with associated shortness of breath, racing heart, and belching. The patient denied nausea, emesis, diaphoresis, and loss of motor/sensory function. The past medical history was significant for type II diabetes, tobacco use, obesity, and hyperlipidemia. The patient's body mass index (BMI) was calculated to be 35.4 upon admission. Family history revealed heart disease in the mother and a noncontributory surgical history. The patient was normotensive, afebrile, and tachycardic with a pulse of 128 bpm and SpO2 of 94%. Physical exam initially presented without pathologic findings.

**Figure 1:**
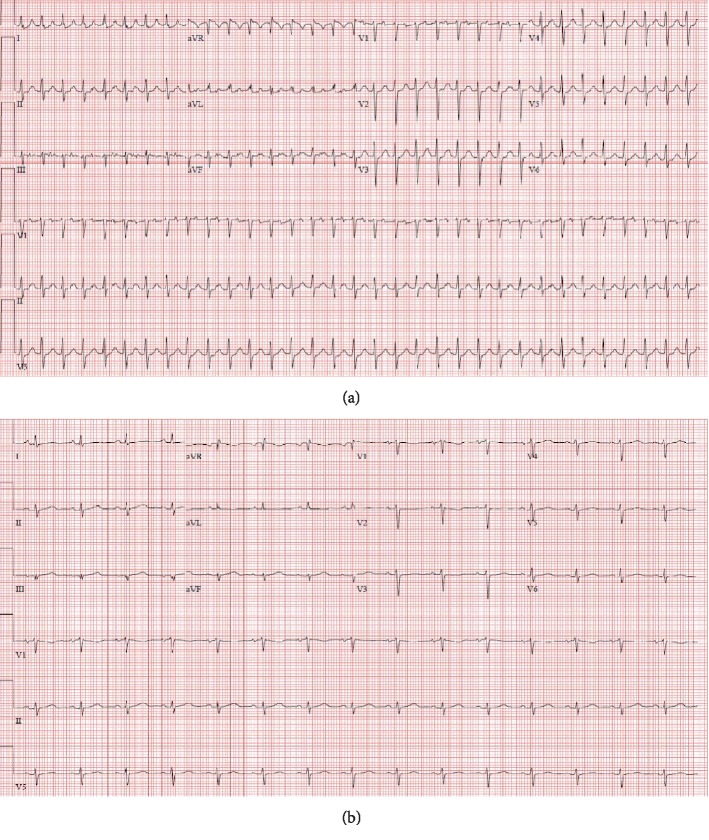
EKG before (a) and after (b) pericardial lipoma resection. (a) Findings: supraventricular tachycardia (197 bpm) with low voltage QRS. (b) Findings: normal sinus rhythm (91 bpm).

**Figure 2:**
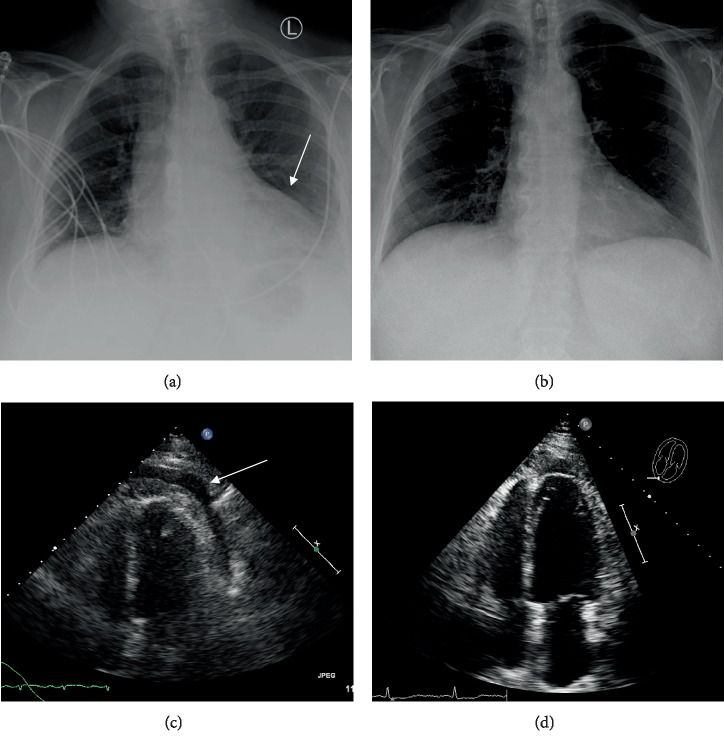
CXR/TTE before (left) and after (right) pericardial lipoma resection. (a) Findings: enlarged cardiac silhouette with epicardial fat pad sign. Impression: cardiomegaly with mild left basilar atelectasis. (b) Findings: the cardiomediastinal silhouette appears WNL. No evidence of pulmonary vascular congestion. Impression: no acute process. (c) Findings: moderately sized pericardial effusion presents circumferentially around the entire heart. (d) Findings: no evidence of cardiac tamponade and minimal remnant pericardial effusion.

**Figure 3:**
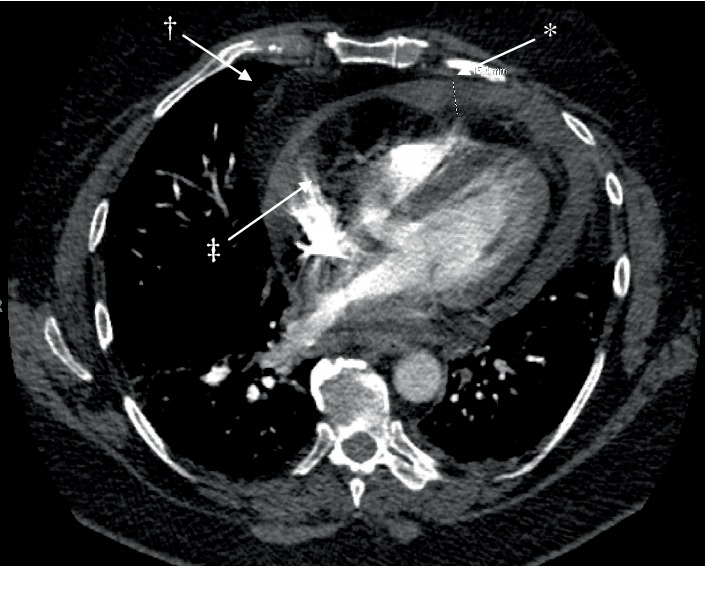
CT pulmonary angiography. Findings: ^∗^pericardial effusion: 15.4 mm (shown). ^†^pericardial mass; ^‡^epicardial fat.

In light of tachycardia, multiple risk factors, and pleuritic chest pain, a pulmonary embolism workup ensued. Electrocardiogram (EKG) showed sinus tachycardia of 139 bpm (Figure 1(a)), and chest X-ray indicated cardiomegaly (Figure 2(a)). Subsequent blood work demonstrated a negative D-dimer of 0.3 ug/mL with unremarkable thyroid-stimulating hormone (TSH) and serial troponins. Additional tests showed an elevated white blood cell (WBC) count of 23.8 K/uL and lactic acid of 2.3 mmol/L (Table 1). A computerized tomography scan for pulmonary embolism (CT/PE) was then performed which displayed pericardial effusion, patchy bilateral pulmonary infiltrate, and pleural effusions (Figure 3). The setting of tachycardia, leukocytosis, and pulmonary infiltrate initiated sepsis protocol. Based on abnormal EKG findings and clinical presentation, the patient was given ketorolac with colchicine for concern of potential pericarditis, as well as azithromycin and ceftriaxone for concern of potential bilateral atypical pneumonia. The patient was then transferred to the intensive care unit (ICU), cardiology was consulted, and a STAT transthoracic echocardiogram (TTE) was ordered.

**Figure 4:**
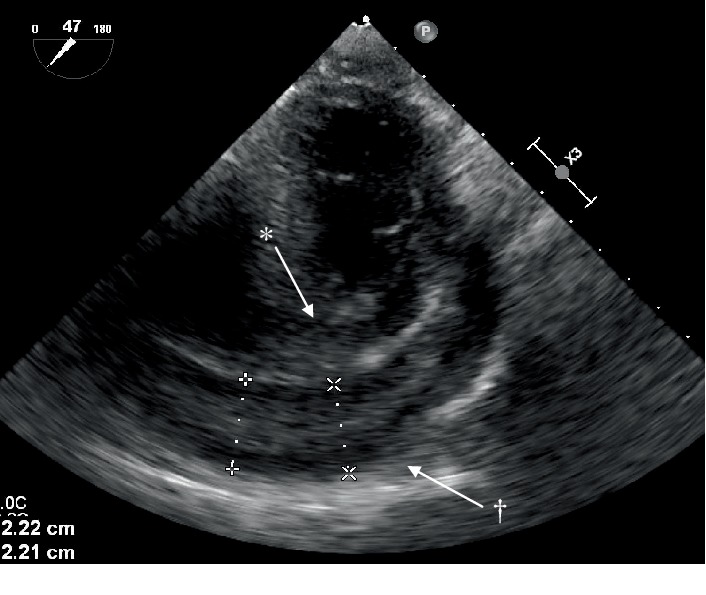
Transesophageal echocardiogram. Findings: ^∗^right ventricular diastolic collapse with ^†^pericardial effusion.

Transthoracic echocardiogram findings were limited and failed to show cardiac tamponade or visualization of the pulmonic valve (Figure 2(c)). Given the patient habitus and unknown underlying tumor, the TTE was suboptimal. Later that night, the patient's tachycardia increased to over 200 bpm, prompting cardioversion. An initial dose of 10 mg intravenous (IV) diltiazem returned the patient to sinus tachycardia temporarily but rebounded to supraventricular tachycardia (SVT). A dose of metoprolol 5 mg IV successfully cardioverted her to sinus tachycardia. The patient was then further stabilized with oxygen and pantoprazole. Given the inconclusive TTE, the patient proceeded to transesophageal echocardiogram (TEE) after stabilization to reevaluate a potential mass and evidence of tamponade. The TEE displayed right ventricular collapse, right atrial collapse, large pericardial effusion, evidence of tamponade, and an echogenic mass in the pericardial effusion (Figure 4). Cardiothoracic surgery deemed the patient a candidate for a pericardial window procedure to treat the effusion and tamponade. While a cardiac MRI was briefly considered, the evidence of tamponade and recurrent SVT prompted immediate surgical intervention rather than additional imaging studies.

The surgical procedure unsheathed a large 10 × 15 cm lipoma in the retrosternal space. This mass was excised and sent to pathology for analysis. Underneath the prepericardial pad, a bulging pericardium was exposed. A small incision in the pericardium expressed 400 mL of dark serosanguinous fluid from around the heart which was sent for cytology, culture, and cell count (Table 2). During surgery, the pericardium was noted to be thickened and inflamed; thus, a 3 × 3 cm biopsy was sent for histologic analysis. The epicardium was also noted to have chronic and acute inflammatory changes. A chest tube was then inserted, and the patient was taken to recovery after extubation.

**Figure 5:**
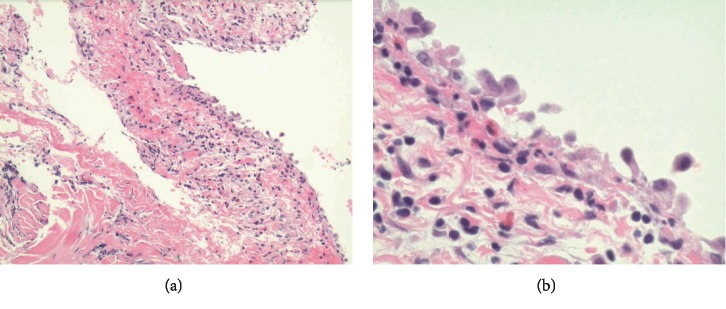
Pericardial biopsy on low power (a) and high power 40x (b). Findings: inflamed hemorrhagic pericardium with lymphocytic involvement.

**Figure 6:**
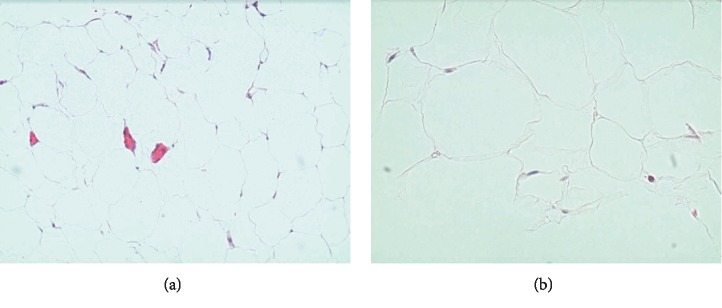
Pericardial lipoma biopsy on low power (a) and high power 40x (b). Findings: benign adipocytes consistent with lipoma. Negative for malignancy.

Pericardial biopsy demonstrated fibroadipose tissue consistent with an inflammatory pericarditis (Figure 5) and benign adipocytes consistent with pericardial lipoma (Figure 6). The pericardial fluid cytology was negative for malignancy and failed to culture acid-fast organisms. Fluid amylase, glucose, protein, triglycerides, and cholesterol were all within normal limits (Table 2). Effusion lactate dehydrogenase (LDH) was greatly elevated at 1,324 IU/L. Given the lab values, etiology was deemed to be an inflammatory pericarditis.

Postoperatively, the patient was initially unremarkable and transferred to the surgical ICU. Later that night, the patient developed new onset atrial flutter status post pericardial window and was placed on diltiazem for rate control. After insufficient rate control, sotalol was added to the treatment regimen along with apixaban for anticoagulation due to the patient's CHADSVASC score of 3. The patient was stabilized in the ICU, and SVT was managed until stabilization for 6 days. Colchicine and ibuprofen were prescribed as initial treatment for the patient's inflammatory pericarditis. Repeat echocardiogram showed no evidence of tamponade and minimal remnant pericardial effusion (Figure 2(d)). The patient's chest tube was removed, and she was discharged postoperatively on day 6 with apixaban 5 mg, colchicine 0.6 mg, diltiazem 30 mg, and sotalol 80 mg. The patient was instructed to follow up with outpatient cardiology to monitor recovery and manage her new postoperative arrhythmia.

## 3. Discussion

Cardiac lipomas are typically asymptomatic, benign heart tumors composed of mature adipocytes void of cellular atypia originating from the subendocardium [5]. However, these lipomas can turn symptomatic in rare circumstances when allowed to grow sufficiently substantial in size. Large cardiac lipomas can cause a wide spectrum of symptoms such as chest pain, exertional dyspnea, arrhythmia, syncope, and lower extremity edema depending on their size and location [3]. Reports have also implicated these neoplasms as causes of sudden death [6]. Pericardial lipomas are an especially rare subset of cardiac lipomas and can present with unique complications due to their location.

**Figure 7:**
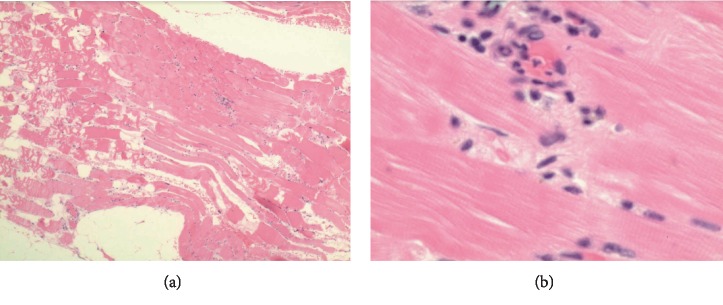
Cardiac biopsy on low power (a) and high power 40x (b). Findings: Lymphocytic infiltration into cardiac muscle, suggestive of myocarditis.

Typically, pericardial lipomas are asymptomatic, with incidental findings found on computerized tomography (CT) or magnetic resonance imaging (MRI) [3]. It is in rare circumstances that these lipomas present symptomatically and even more unusual to be substantial enough to induce cardiac tamponade. In this case, there were two leading theories regarding causalities of cardiac tamponade: cardiac compression by pericardial lipoma leading to hemodynamic compromise or inflammation resulting in fibrosis and pericardial fluid accumulation due to extracellular matrix destruction and increased vascular filtration [7]. For this patient, biopsies of the cardiac muscle and pericardium confirmed that cardiac tamponade was induced by activation of proinflammatory markers from the patient's giant 10 × 15 cm anterior pericardial lipoma. Lymphocytic infiltration of the cardiac muscle was negative for fibrosis, suggesting acute phase presentation of disease (Figure 7). Had intervention been further delayed, myocarditis may have ensued.

In the instance of a suspected cardiac tumor, transthoracic echocardiogram is most commonly the initial method of imaging investigation due to its ease of access and noninvasive application [8]. With 90% sensitivity and 95% specificity, echocardiography provides dynamic evaluation when detecting cardiac tumors with capacity for physiologic characterization of consequences [9]. However, transesophageal echocardiography may provide a better, unobstructed view of the heart as it is not limited by an acoustic window and is uninterrupted by the chest wall or lungs [10]. In nonemergent situations, enhanced evaluation and confirmatory testing for a suspected cardiac lipoma can be achieved with cardiac computerized tomography (CCT) and cardiac magnetic resonance (CMR) [10, 11]. A positron emission tomography and computed tomography (PET-CT) scan should also be obtained when attempting to accurately identify the presence or absence of metabolic activity within a suspected mass and reveal possible sites of lesion metastasis. Ultimately, diagnosis of a nonspecific cardiac mass requires specimen biopsy to understand its composition and origin.

Upon diagnosis of a pericardial lipoma, surgical excision by median sternotomy is indicated as both the therapeutic and diagnostic course of treatment [8]. With the incidence of postoperative atrial fibrillation after major thoracic surgery being not uncommon at >30% [12, 13], monitoring for postoperative arrhythmias (usually atrial fibrillation or flutter) has become common practice for these procedures [14]. However, this elevated awareness does not mean arrhythmias are of little consequence, as resultant hemodynamic compromise and thromboembolism are serious contributors to morbidity and mortality in these patients [15]. Prophylactic drug therapy is currently the best method for avoiding postoperative arrhythmic complications and should be considered when managing a patient after the excision of a pericardial lipoma [16]. Sotalol has demonstrated efficacy as a prophylactic agent for postoperative arrhythmias when compared to placebo, with amiodarone being useful as an additional agent in high-risk patients [17]. Alternatively, recent evidence suggests that preservation of the anterior cardiac fat pad during lipoma resection may also reduce the risk of postoperative arrhythmias [18]. While this modified technique does have the potential to reduce the incidence of postoperative arrhythmia, it is uncertain as to whether it would have significantly decreased the likelihood of postoperative arrhythmia and concomitant life-long medical therapy in this case.

## 4. Summary

Chest pain, while a common presentation, can be benign or potentially fatal. Casting a wide differential while working up chest pain, rather than defaulting judgment to solely common presentations, can have a significant impact on patient care. While patients with benign pericardial tumors carry a good prognosis, acute irritation can result in long-term complications and even fatality if not recognized and managed correctly.

## Figures and Tables

**Table 1:** Preliminary laboratory values.

CBC labs	Patient's value	Reference range	Additional tests	Patient's value	Reference range
WBC	23.8	3.8–10.6 K/uL	BUN	14	10–25 mg/dL
RBC	4.93	4.15–5.55 M/uL	Cr	.77	<1.03 mg/dL
Hg	14.2	12.0–15.0 g/dL	D-dimer	.3	0–0.50 ug/mL
Hct	37.2	36–46%	Lactic acid	2.1	0–2.1 mmol/L
Plt	390	150–450 K/uL	Troponin	<40, <40	0–50 ng/L

**Table 2:** Fluid analysis consistent with inflammatory pericarditis.

Test	Patient's value	Reference range	Tests/culture	Patient's value	Reference range
Albumin	2.6	0–4.6 g/dL	TAG	67	0–200 mg/dL
Amylase	16	0–100 IU/L	RBC	67,481	Cu/mm (N/A)
Cholesterol	125	0–200 mg/dL	WBC	4,228	Cu/mm (N/A)
Glucose	86	0–100 mg/dL	Bacteria culture	Negative	N/A
LDH	1,342	0–220 IU/L	Fungal culture	Negative	N/A
Protein	67	<8.2 g/dL	AFB culture	Negative	N/A
